# ID 285 - JAV (Jornada Assistencial de Valor) RARAS: análise de custos utilizando TDABC para pacientes com distrofia muscular de Duchenne em serviço de atenção em doenças raras

**DOI:** 10.5327/2237-9622.2025.v34s1.285

**Published:** 2025-11-25

**Authors:** Camila Correa Azevedo, Myrianne Gilsara Soares e Barbosa, Marilaine Menezes Ferreira, Marcela Mahado, Marcelo Nita, Luana Lopes, Thiago Godoy, Têmis Félix

**Keywords:** custeio, avaliações econômicas, processos assistenciais, impacto orçamentário

## Abstract

**Introdução:**

O JAV-RARAS integra o projeto da Rede Nacional de Doenças Raras (RARAS), focando a análise da Jornada Assistencial de Valor (JAV) de pacientes com doenças raras no Brasil. O objetivo é registrar essas doenças, definir parâmetros de valor para os pacientes e avaliar a relação custo-efetividade, permitindo, dessa forma, analisar e otimizar a alocação de recursos em diversas regiões do País. Os dados foram coletados em serviço de atenção em doenças raras, localizado em Salvador, Bahia. Este estudo tem como objetivo específico quantificar e descrever os custos relacionados à trajetória assistencial de pacientes com distrofia muscular de Duchenne (DMD). Ele se integra ao projeto JAV-RARAS, que faz parte da Rede Nacional de Doenças Raras, utilizando a metodologia de (TDABC).

**Método:**

A pesquisa que utiliza a metodologia TDABC consiste na coleta de informações relacionadas ao diagnóstico, tratamento e acompanhamento de pacientes no âmbito do estudo JAV-RARAS. Inicialmente, foi realizado um mapeamento da trajetória assistencial no centro envolvido, abrangendo cinco doenças raras, com a quantificação do tempo e dos recursos utilizados nas etapas de diagnóstico, tratamento e acompanhamento. Os custos diretos foram obtidos por meio de entrevistas e análise de registros administrativos de profissionais de saúde, em centros espalhados pelo País. A análise incluiu a identificação das atividades na trajetória do paciente com DMD, a alocação de recursos, o cálculo dos custos por unidade e a medição da duração das atividades no serviço de atenção em doenças raras.

**Resultados:**

O custo médio anual para um paciente em tratamento de DMD é de R$ 198.905,34, com a maior parte dos gastos concentrada em medicamentos. A distribuição dos custos inclui: recursos humanos (R$ 253,20), materiais (R$ 541,74), medicamentos (R$ 197.240,40) e exames (R$ 870,00). Todo o investimento anual é destinado exclusivamente ao tratamento da doença. Quanto à origem dos recursos, em torno de 82,5% são provenientes do serviço, enquanto 1% é financiado pela indústria e 10% pelo Sistema Único de Saúde (SUS) em outras localidades.

**Conclusão:**

Este estudo ressalta a relevância de levantar e analisar os custos e o acesso a intervenções terapêuticas no tratamento da DMD. A predominância das despesas anuais atribuídas a medicamentos evidencia a necessidade de uma compreensão mais profunda dos fatores que impactam esses custos. A exploração de alternativas terapêuticas mais eficazes pode potencialmente reduzir os custos em longo prazo e aprimorar os resultados clínicos dos pacientes.

